# Characterization of a Radiofluorogenic Polymer for Low-Energy Electron Beam Penetration Depth Visualization

**DOI:** 10.3390/polym14051015

**Published:** 2022-03-03

**Authors:** Magdalena Maria Skowyra, Christina Ankjærgaard, Liyun Yu, Lars René Lindvold, Anne Ladegaard Skov, Arne Miller

**Affiliations:** 1 Department of Health Technology, Technical University of Denmark, Frederiksborgvej 399, 4000 Roskilde, Denmark; cank@dtu.dk (C.A.); lali@dtu.dk (L.R.L.); armi@dtu.dk (A.M.); 2 Department of Chemical and Biomedical Engineering, Technical University of Denmark, Søltofts Plads 228A, 2800 Kgs. Lyngby, Denmark; lyyu@kt.dtu.dk (L.Y.); al@kt.dtu.dk (A.L.S.)

**Keywords:** low-energy electron beam, fluorescence, polymer dosimeter, radiofluorogenic, 3D dosimetry

## Abstract

Low-energy (80–300 keV) electron beam accelerators are gaining in importance in the radiation processing industry due to their ease of use and wide range of applications (e.g. product surface sterilizations or polymer curing and cross-linking). Due to their very low penetration depth (tens to hundreds of microns), currently used film dosimeters exhibit dose gradients over their thickness and do not resolve the dose response in the first microns of the irradiated material. Hence, the surface dose, defined as the dose in the first micron D_µ_, cannot be measured directly. This study presents a polymer material as a dosimeter candidate for high-dose low-energy electron beam irradiations. The readout of the dose-dependent fluorescence intensity, originating from a pararosaniline dye reaction when irradiated, is measured using fluorescence microscopy. So far, no in-depth characterization of the material has been performed, leaving the stability and fluorescence properties of the material not fully optimized. We describe the improvements in polymer composition and the fabrication method, and characterize the material properties in terms of the thermal stability, glass transition temperature, refractive index, hardness, rheological behavior, and water affinity. All of these create a complex set of requirements a polymer needs to fulfill to become an effective dosimeter when measuring using confocal microscopy. The fluorescence readout procedure will be addressed in further studies.

## 1. Introduction

Having its beginnings in the 1950s, electron beam (e-beam) industrial processing has been developing rapidly and used extensively in many areas of industry, where there is a need for enhancement of the physical and chemical properties of materials or a reduction of undesirable contaminants [1]. Low-energy electron beams, which are typically of energies ranging between 80 and 300 keV [2], have found several applications in the radiation processing industry, starting from polymer curing [3], cross-linking of coatings and inks [4] and polymer functionalization by grafting [5], through decontamination of packaging materials [1], up to sterilization of surfaces [6,7] and pharmaceutical components [8]. Low-energy e-beam accelerators offer a number of advantages, such as (1) electrical energy conversion of the order of 70% [9], (2) high dose rates (~100 kGy/s), allowing rapid processing [1], (3) radiation protection in a form of self-shielded equipment [1], (4) environmental benefits, as they reduce the emission of volatile organic compounds and air pollutants [10], and (5) low costs [1].

However, low-energy electrons are able to penetrate matter only up to a few hundred micrometers [2], which in fact becomes challenging in terms of absorbed dose determination. The dosimetry requirements are especially rigorous when performing the surface sterilization process of, for example, pharmaceutical or medical products [11]. There, only the external surfaces of, for example, pre-sterilized tubs (of vials or syringes) should be irradiated before they can enter an aseptic filling area. In this way, the production of radiolysis products within the volume of a tub is avoided [12].

Several dosimetry systems are used in the radiation processing industry, such as alanine-EPR [13], calorimeters [14] or radiochromic dye films [13,15]. They are all excellent tools for high-dose measurements (~kGy). However, at low electron energies, dose gradients are introduced over the thickness of a dosimeter, and the measured average doses will depend on the chosen system [16]. In addition, they are not able to measure the dose in the first microns of irradiated material, the so-called surface dose. Thus far, the surface dose has been extrapolated from obtained depth–dose curves and corrected to the average dose in the first micrometer—D_µ_ [16]. Schuster et al. [17] proposed a thin dosimetric spray-coating material for surface dose measurements of complex surface geometries (exemplified by PET bottles). The concept relies on a readout of the dose-dependent luminescence decay time of an inorganic phosphor under near-infrared excitation. However, fading of the phosphor was a limiting factor that this dosimetry system so far has not been able to overcome, and hence, the challenge of directly measuring the dose in the first micrometer of a material is still to be addressed.

Here, we investigate a new type of material that could be applied as a low-energy electron beam dosimeter, focusing on surface dose verification. As a starting point, a radiochromic and radiofluorogenic polymer dosimeter developed by Bernal-Zamorano et al. [18,19,20], which was initially intended to work as a dosimeter for the medical dosimetry dose range (~Gy), was selected. However, industrial radiation processing applies doses of the order of tens or even hundreds of kGy, so the material had to be appropriately modified. Furthermore, the material’s composition, its mechanical, optical and thermal properties, hydrophilicity as well as the photocuring process had not yet been fully optimized or characterized. The system previously developed by Skowyra et al. [21] was a proof of concept where the solid state polymer material responded to kilogray radiation doses of low-energy electron beam irradiations by its change in fluorescence intensity, measured using an epi-fluorescence widefield microscope. However, issues related to the background fluorescence signal, shelf life and environmental stability of the material prompted further investigation into establishing and demonstrating suitable material requirements before optimizing the readout technique into confocal laser scanning microscopy.

In this paper, we investigate the influence of the polymer material’s composition on the properties of the material. This includes the polymer matrix and the optimal solvent for the radiation-sensitive dye. We present the material’s properties in terms of its thermomechanical behavior, particularly the decomposition process and glass transition temperature, as well as hardness and rheological studies. Additionally, the optical quality of the polymer, with a focus on refractive index measurement, was determined, and for fluorescence imaging purposes, the water affinity of the material was evaluated. Finally, we show the absorbance and fluorescence response spectra of pararosaniline leuco-dye in both a solution and solid material, and demonstrate the spectral response of the material irradiated with a low-energy electron beam.

## 2. Materials and Methods

### 2.1. Composition

The dosimeter material consisted of (1) host polymer–PPGDA (poly(propylene glycol) diacrylate, 1.12 M, Sigma-Aldrich, Darmstadt, Germany, CAS: 52496-08-9, inhibitors: 100 ppm BHT and 100 ppm MEHQ); (2) photoinitiator–TPO (diphenyl (2,4,6-trimethylbenzoyl) phosphine oxide, 28 mM, Sigma-Aldrich, Germany, CAS: 75980-60-8); (3) leuco-dye (4′,4′,4″-triamino-triphenylaceto-nitrile, 6.89 mM, NCK A/S, Farum, Denmark); and (4) acetonitrile (0.99 M, Sigma-Aldrich, Germany, CAS: 75-05-8, max. 0.005% H_2_O).

The solvents tested for solubility were acetone (CAS: 67-64-1, ≥99.5%), acetonitrile (CAS: 75-05-8) and ethanol (CAS number: 64-17-5, ≥99.9%) supplied by Sigma-Aldrich (Merck, Germany) and 2-ethoxy ethanol (CAS: 110-80-5, ≥98.5%) provided by Fluka Chemie (Buchs, Switzerland). The concentration of the dye solution was 20 mg/mL.

### 2.2. Fabrication

The leuco-dye, the selected solvent (acetonitrile) and the host polymer were mixed at room temperature using a magnetic stirrer in a closed, UV-blocking glass vial for 30 min. After that, the photoinitiator was added, and the solution was mixed for another half an hour. The mixed composition was then placed in a refrigerator at 4 °C until the photocuring was carried out to lower the oxygen diffusion and avoid a viscosity increase [22]. The samples were prepared by means of a photopolymerization reaction using a LED lamp of a 385 nm wavelength as described in [21]. Three different sample types were manufactured: (1) cuboids of dimensions 3 × 3 × 45 mm, (2) cuboids of dimensions 1 × 3 × 45 mm and (3) cylinders 10 mm in diameter and 3 mm in thickness. The samples were cured in silicone molds [21] of corresponding shapes with a plastic foil placed on the top surface to block oxygen inhibition during the reaction [23]. The illumination time was 5 min, with the intensity of the UV light measured to be 15.0 ± 0.3 mW/cm^2^. Unless otherwise stated, the uncertainties throughout the performed measurements were given at one standard deviation (*k* = 1).

Two resins for 3D-printed molds were tested as alternatives for the silicone mold: (1) traditional resin (black, ELEGOO standard [24]) and (2) acrylonitrile butadiene styrene (ABS)-like resin (transparent, ELEGOO ABS-like [25]). The compositions of the resins were: epoxy resin (50%), hexamethylene diacrylate (40%) monomer, N-(dimethylcarbamoyl) glycine (5%) and hydroxycyclohexyl phenyl ketone (5%) photoinitiator. The molds were 3D printed using stereolithography (SLA) technology.

### 2.3. Irradiation Process

Irradiations were carried out using a low-energy electron beam accelerator at the Risø High Dose Reference Laboratory (e-beam, EBLab-200, COMET AG, Wünnewil-Flamatt, Switzerland), maintaining a 15-m/min conveyor speed and 15 mm air gap between the sample surface and electron beam window at, laboratory conditions with an average temperature of 21 °C and a humidity level between 30 and 50%. The samples were placed on a polystyrene backplate and fastened with tape to ensure surface evenness. The irradiation process parameters used throughout for these measurements are presented in Table 1.

### 2.4. Thermogravimetric Analysis (TGA)

In order to establish the thermal stability of the polymer material, thermogravimetric analysis was performed. The measurements of weight loss versus temperature were carried out on a Discovery TGA (TA Instruments, New Castle, DE, USA). The TGA data also helped in selecting the experimental conditions for the differential scanning calorimetry measurements. 10 mg of the polymer (top surface part) was placed in a platinum pan and heated in a nitrogen atmosphere from room temperature to 900 °C, with a heating rate of 10 °C/min. The readout of the sample weight versus temperature was performed every 0.01 s. The analysis was performed on both non-irradiated and irradiated samples (120 keV e-beam, 30 kGy), and the results were analyzed by TRIOS software (TA Instruments, New Castle, DE, USA). The accuracy of the weight determination was <±0.1% of a measured value.

### 2.5. Differential Scanning Calorimetry (DSC)

Differential scanning calorimetry (DSC) was conducted to determine the phase transitions of the polymer by measuring the heat flow produced in a sample as a function of the temperature. Particularly important was the determination of the glass transition temperature (T_g_), as this indicates what physical properties of the polymer can be expected in a specified range of temperatures and how they will later influence the dosimetric properties of the material. A total of 2–5 mg of the polymer was cut into small pieces and placed in the T_zero_ aluminum hermetic pan. The masses of the pieces were determined with the uncertainty of ±0.01 mg. Both the non-irradiated and irradiated material was tested. DSC measurements were performed in a nitrogen atmosphere on the Discovery TGA with a heating and cooling rate of 10 °C/min. Each measurement consisted of a first heating step from −90 °C to 200 °C (isothermal at −90 °C for 5 min), a cooling step from 200 °C to −90 °C (isothermal at −90 °C for 5 min) and finally a second heating step from −90 °C to 300 °C.

The glass transition temperature was measured from DSC curves as the endothermic transition from the amorphous glassy state into the viscous rubbery state [26] by means of the second heating scan. T_g_ was determined using TRIOS software (TA Instruments, New Castle, DE, USA) as the midpoint (i.e. halfway point) temperature of the jump in heat capacity in the chosen temperature range [27]. The temperature accuracy of the equipment was ±0.1 °C, and the temperature precision was ±0.05 °C.

### 2.6. Refractive Index

For the purpose of verifying the homogeneity of the material and finding a refractive index matching medium for fluorescence microscopy readouts, optical coherence tomography (OCT) was used. The refractive index (RI) measurements of a cured polymer sample were measured using Spectral Domain Optical Coherence Tomography (Telesto-II, Thorlabs Inc., Newton, NJ, USA) by applying the optical path shifting method [28,29] at a wavelength of 1310 nm. At this wavelength, no absorption of light was observed by the polymer material. Only when the optical path length of the sample and reference arm match, the observed interference signal reach a maximum. Hence, it can be used to measure the distance precisely. First, an image of the reflection coming from the scanning stage (glass) was acquired. While keeping the distance between the probe and the scanning stage fixed, the cuboid sample (Type 2) was placed on the scanning stage, and another image was acquired. In OCT measurements, the measured value of the thickness does not present its real thickness but its optical thickness OT [29]:

(1) OT=n×T 

where *n* and *T* are the refractive index and the physical thickness of the sample, respectively. After the sample placement, the position of the scanning stage shifts down due to imaging of material with a refractive index larger than 1, and that optical shift *OS* can be expressed as follows [29]:

(2) OS=OT−T 

Consequently, the refractive index *n* of the sample was calculated using the following equation:

(3) $$n=\frac{OS}{T}+1$$

The RI of three samples was measured, and the uncertainty of one standard deviation (k = 1) of the result was determined. The physical thickness of the samples was measured using a Millitron Meter (Mahr Feinprüf, Germany, Type 5312340 230 V) with an accuracy of ±1 µm.

### 2.7. Hardness

Measurements of the hardness of the polymer material were conducted in order to test if additional post-processing high-temperature heating was needed. The hardness of the polymer material was measured using a Shore A durometer (AD-300, Checkline Europe, Enschede, The Netherlands). Cured, non-irradiated samples (Type 2), as well as samples heated in an oven at 180 °C for 10 min, were tested. Six samples, each approximately 1 mm thick, were stacked on top of each other to fulfill the minimal thickness requirement of 6 mm for the measurement [30]. Five measurements of both sample types were carried out, each with a time duration of 15 s.

### 2.8. Rheology

Rheological studies of the polymer material were performed in order to determine its viscoelastic properties that contribute to the optimal dosimetric properties. The viscoelastic properties of the polymer material were tested using a rheometer (DHR-1, TA Instruments, New Castle, DE, USA) with a 20-mm parallel plate (Peltier plate steel 106669), a gap of the sample’s thickness (1 mm) and temperature of 25 °C. Some 1-mm thick cylinder samples were cut out of cuboid samples (Type 2). Amplitude sweep tests were performed for three different samples by applying an angular frequency of 10 rad/s in the strain region from 0.1 to 10% in order to determine the linear viscoelastic region of the polymer material. For the chosen strain of 0.25%, the frequency sweep test of those samples was run in the angular frequency region from 100 to 0.1 rad/s. In addition, a temperature sweep measurement was performed by applying 0.25% strain at an angular frequency of 10 rad/s in the temperature range of 10–100 °C. A frequency sweep test was also run for samples irradiated with a low-energy e-beam of energies 80, 120 and 200 keV and a dose of 50 kGy, applying 0.25% strain in the frequency region from 100 to 0.1 rad/s.

### 2.9. Water Affinity

The water affinity of the polymer material was tested in order to determine the material swelling properties and the possible use of a water immersion objective for fluorescence imaging. Water contact angle (WCA) measurements were conducted on an OCA20 Contact Angle System (DataPhysics Instruments GmbH, Filderstadt, Germany) using a sessile drop technique at room temperature. A 6 µl droplet of MilliPore water was dispersed through a needle and placed on the surface of the sample. The non-irradiated and irradiated (200 keV, 30 kGy) samples’ top, bottom and side surfaces were tested. The WCA was determined using SCA20 software (DataPhysics Instruments GmbH) as an average value of three measurement points on each of the sides.

### 2.10. Absorbance and Fluorescence Spectra

The absorbance and fluorescence spectra were measured using a Tecan Spark M10 multimode plate reader (Tecan Trading AG, Männedorf, Switzerland) with a 12-well plate (VWR Tissue Culture Plates). The absorbance spectra were measured as the optical density (OD) in the wavelength range of 300–800 nm with a wavelength step size of 2 nm. The fluorescence (emission) spectra were recorded in the wavelength range of 560–800 nm with a step size of 1 nm, after excitation with 555 nm light of a 5 nm bandwidth. The fluorescence signals were quantified as relative fluorescence units (RFUs) while keeping a constant gain of 100 (the amplification factor for the photomultiplier).

For this, 0.5% *w*/*v* of the leuco-dye was dissolved in the selected solvent (acetonitrile) and divided into two parts: (1) a non-irradiated liquid solution (kept in laboratory conditions up to the measurement time) and (2) an irradiated liquid solution (200 keV e-beam, 30 kGy). The liquid solution was irradiated in a thin (0.5 mm) metal petri dish. The solid samples were prepared as described in Section 2.2 and divided into: (1) a non-irradiated solid and (2) an irradiated solid (200 keV e-beam, 30 kGy). Additionally, the absorbance and fluorescence spectra of polymer material, irradiated with a low-energy e-beam of 80 keV and 5 doses (15, 20, 30, 40 and 50 kGy), were measured. The spectra were normalized to the absorbance and fluorescence peak value of the 50 kGy sample.

## 3. Results

### 3.1. Dosimeter Composition

#### 3.1.1. Solubility of the Dye

Pararosaniline leuco-dye, used as a radiation-sensitive medium, needs to be fully dissolved before it is mixed with other chemicals. This step is particularly important in order to ensure the uniformity of the samples and their later response to the radiation dose, measured as fluorescence intensity values. Accumulation of undissolved dye might also impair the fluorescence readouts by introducing shadowing or scattering effects. The composition developed by Bernal-Zamorano et al. [19] used ethanol as a solvent. However, our observations indicated that the dye was sparingly soluble, which is also in agreement with the literature values of triphenylmethane dye solubility in ethanol [31]. The dye formed a precipitate and rapidly changed its color once dissolved in ethanol. The dye is reported to be very soluble in solvents like diethyl ether, pyridine, chloroform and benzene [31], but none of these were considered here due to their toxicity. The commercially used Risø B3 radiochromic film [32], which contains the same radiation-sensitive dye as our dosimeter material, uses 2-ethoxy ethanol as a solvent in the fabrication process, and hence it was also tested. Additionally, acetone and acetonitrile were tested due to their lower toxicity and versatility.

All the tested solvents—2-ethoxy ethanol, acetone and acetonitrile—successfully dissolved the required amount of dye. However, the 2-ethoxy ethanol solution very quickly changed its color to magenta and was not further tested, which was also due to its toxicity. Acetone and acetonitrile solutions, after mixing with the rest of the composition and being stored in the refrigerator for 5 days, showed much less coloration. However, the acetone solution started to precipitate after a few days. Due to acetone being more volatile [33] and less polar than acetonitrile [34], acetonitrile was chosen as a solvent for the composition.

#### 3.1.2. Host Polymer: PEGDA vs. PPGDA

The polymer matrix, which is formed by cross-linking of a diacrylate host polymer, must provide stiffness for the embedded leuco-dye so that it can undergo a radiofluorogenic reaction and efficiently emit the fluorescence signal. The environment of the fluorescent molecule should hinder the mobility of the benzene groups of the leuco-dye. The fluorescence is known to be favored in a more viscous medium [35,36,37], as the energy released by other means, such as vibrations and rotation, becomes suppressed. Previous systems [19,21] employed poly(ethylene glycol) diacrylate (PEGDA) as the host polymer with the addition of 2-hydroxyethyl methacrylate (HEMA) as an additional polymer for increased mechanical stability. However, we observed that the polymer prepared using that composition was very sensitive to UV light, and the coloration process of the dye would start even in daylight conditions after a few minutes. Moreover, HEMA seemed to introduce an increase in the autofluorescence signal seen in the fluorescence spectra, which was due to the hydroxyl group present in the backbone that may undergo a deprotonation process in the presence of amino groups in the leuco-dye structure. Additionally, in order to limit the rotational freedom of the phenyl groups of the dye and improve the rigidity, bulkier substituents should be present in the polymer chain [38], as in the case of poly(propylene glycol) diacrylate (PPGDA), where a single methylene group is introduced. However, polymers with longer side chains are not recommended, since they might favor flexibility and cause a plasticizing effect. The same applies to branched substituent groups—even though they might support the stiffness requirement, the prepolymers were usually extremely viscous, and the cured samples showed restricted diffusion of oxygen, preventing the reaction of the leuco-dye. Hence, the new composition contained PPGDA as a host polymer, which is UV transparent and has good polarity [39], stability and solubility properties [40].

### 3.2. Sample Preparation: Curing Mold

**Figure 1:**
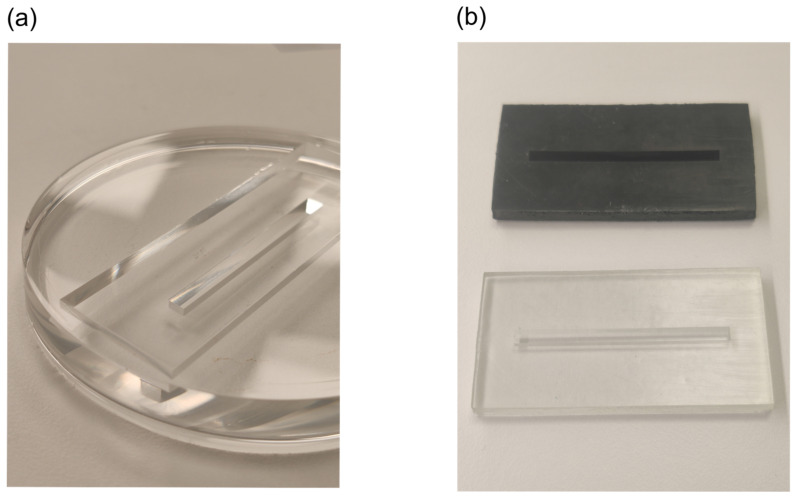
Manufacturing molds: (**a**) silicone mold, (**b**) 3D-printed molds: transparent (bottom) = ABS-like resin; black (top) = traditional resin.

The photopolymerization process from a liquid solution into a solid sample took place in a specially designed silicone mold, like the one shown in Figure 1a. We observed that the lifetime of the silicone mold was limited to approximately 2 months of daily use. After that, the high-intensity UV light used for curing caused damage to the silicone [41], which started interacting with the polymer solution and drastically worsened the optical quality of the sample surfaces. Therefore, we tested two different 3D-printed molds, presented in Figure 1b. Both of them, however, exhibited sticking of the cured solution inside the 3D-printed mold, with no possibility of removal because of the lack of flexibility of the mold. This was probably due to the polymer solution reacting with some part of the residue resin that was not fully cured during 3D printing. The photopolymerization process of polymer samples used UV light of a wavelength close to the excitation wavelength of the printed resin’s photoinitiator. Therefore, it was decided to continue using the silicone mold for the specified lifetime of 2 months, being approximately 100 curing procedures. Nonetheless, other 3D printing resins with higher flexibility and lower hardness could be tested, but this was beyond the scope of this study.

### 3.3. Material Characteristics

#### 3.3.1. Thermal Stability

**Figure 2:**
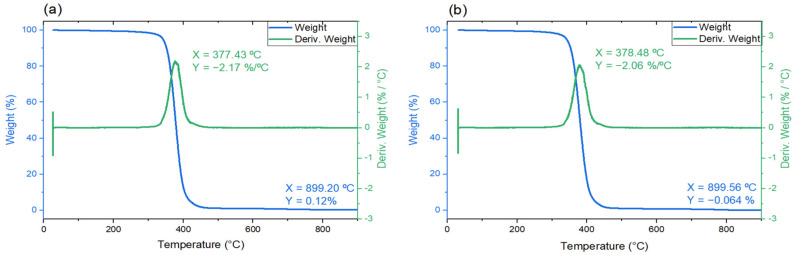
TGA curves of: (**a**) non-irradiated and (**b**) irradiated (120 keV, 30 kGy) polymer material. Blue = weight (%) vs. temperature (°C); green = the derivative of weight with respect to temperature (%/°C). Green coordinates illustrate the maximum of the derivative of weight, and blue coordinates represent the end weight of the tested sample.

TGA measurement of the non-irradiated and irradiated (120 keV e-beam, 30 kGy) samples was carried out to determine the decomposition temperature of the polymer, which was further needed for the selection of experimental conditions (temperature range) for the DSC measurements. The weight loss versus temperature curves for the non-irradiated and irradiated material can be seen in Figure 2a,b, respectively. The thermal treatment caused the mass of the polymer to change continuously, and rapid weight loss of the material started to occur at around 350 °C, followed by a complete, one-step decomposition at around 380 °C for both the non-irradiated and irradiated samples. In the end, after heating to 900 °C, there were no residues left in the crucible, leaving the material fully degraded.

#### 3.3.2. Glass Transition Temperature

**Figure 3:**
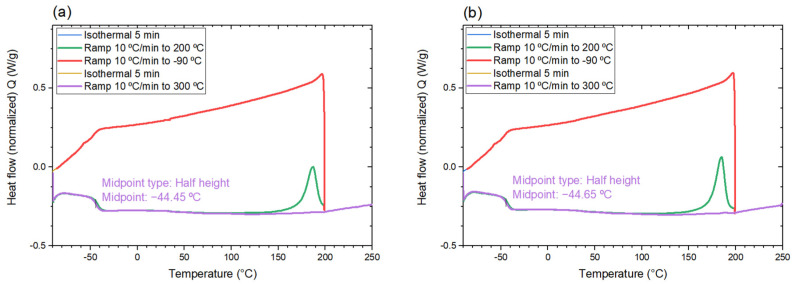
DSC curves of: (**a**) non-irradiated and (**b**) irradiated (80 keV, 30 kGy) polymer material. Green = 1st heating cycle; red = cooling step; violet = 2nd heating cycle. Tg value (midpoint) is presented on the graph in purple.

The measurement of the heat flow versus temperature of the non-irradiated and irradiated (80 keV e-beam, 30 kGy) polymer material can be seen in Figure 3a,b, respectively. The first heating cycle erased the thermal history of the material and left the determination of the glass transition temperature in the second heating cycle more distinct. The calculated T_g_ of both the non-irradiated and irradiated material was around −45 °C, with no changes introduced during the irradiation process. The glass transition temperature of the material is below room temperature, which means that the polymer will be in a rubbery state while working at the intended temperature. Above T_g_, it will remain soft and flexible [38], and because large elastic deformations are possible, the polymer is actually both tougher and more pliable [26]. This allows for a more profound oxygen diffusion [42], which is needed for the reaction of the leuco-dye to proceed. Furthermore, it provides a rigid polymer matrix, which facilitates the fluorescence emission of the dye [35].

An exothermic peak appeared during the first heating cycle for both the non-irradiated and irradiated material, with its maximum at around 180 °C. It could have its origin in some additional high-temperature curing reaction of a residual monomer or a reaction commencing due to free radicals trapped in the polymer matrix [43], since the obtained double-bond conversion was of the order of 80%. In Section 3.3.4, it is tested how a post-manufacturing heating at 180 °C may influence the hardness, and consequently the material stiffness.

**Figure 4:**
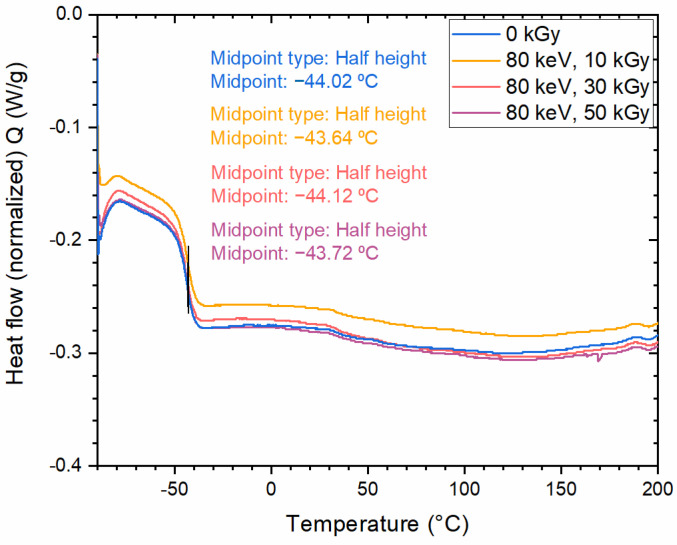
DSC curves: second heating cycle for polymer material irradiated with 80 keV electron beam for 3 doses: 10, 30 and 50 kGy. A curve for non-irradiated polymer (0 kGy) is also presented for comparison.

Additionally, a measurement of the glass transition temperature of the polymer samples irradiated with an 80 keV e-beam and different nominal doses of 10, 30 and 50 kGy was performed. Their second heating cycles are presented in Figure 4. No influence of the irradiation dose on the glass transition temperature was observed, meaning that the polymer material did not undergo any significant chain scission, chain reorganization or cross-linking while exposed to high-dose e-beam radiation. The glass transition temperature is a function of the polymer’s molecular weight [44], and it may be altered after exposure to radiation, which is known to cause chain scission and increase the polydispersity of the molecular weight [45].

#### 3.3.3. Refractive Index

**Figure 5:**
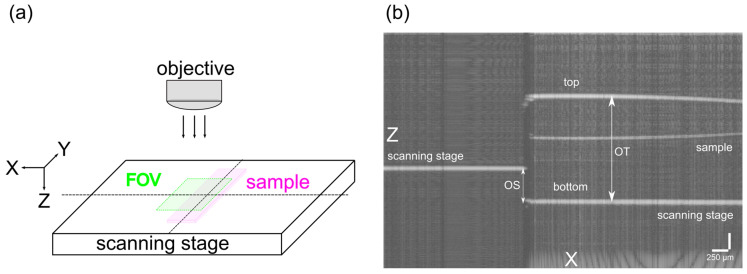
(**a**) The scheme of the OCT readout. Sample (magenta) placed on a scanning stage, and XY field of view (FOV, green) is chosen for the axial profile measurement. (**b**) OCT B-scan of the polymer sample for RI measurement using optical path shifting method. OT = optical thickness of the sample; OS = optical shift of the scanning stage.

The refractive index of a cured polymer sample gives information about what kind of immersion medium has to be used during fluorescence imaging in order to minimize the refractive index mismatch [46]. A mismatch might lead to poor imaging quality, and therefore a correction factor has to be introduced to the axial intensity distribution. A schematic of the readout geometry is presented in Figure 5a, where a sample (magenta) is placed on a scanning stage. The OCT axial scan (B-scan) of the single field of view (FOV) is shown in Figure 5b. The calculated refractive index of the polymer material was *n* = 1.47 ± 0.01. This finding suggests that fluorescence imaging should be performed in an immersion medium of a refractive index between 1.46 and 1.48, (e.g. glycerol) [47] or a fluorescence immersion oil (Type FF, Cargille-Sacher Laboratories). Another possibility would be to correct the introduced axial distortion, caused by the difference between the observed optical thickness and actual thickness, by multiplying the axial sampling by 1.47 (assuming air of *n* = 1 as the surrounding medium). It was also observed that the material was optically clear, and its properties were not dominated by scattering. The thin line visible in the middle of the sample (Figure 5b) might be a reflection of the top of the sample from the glass scanning stage.

However, the refractive index value is dependent on the wavelength [48], a phenomenon known as dispersion. Hence, the value measured at 1300 nm might be different, in this case lower from the value at the excitation wavelength of 555 nm. This discrepancy may be a cause of additional uncertainty introduced due to imaging in a medium with a different refractive index or when correcting for the axial distortion.

#### 3.3.4. Hardness

**Figure 6:**
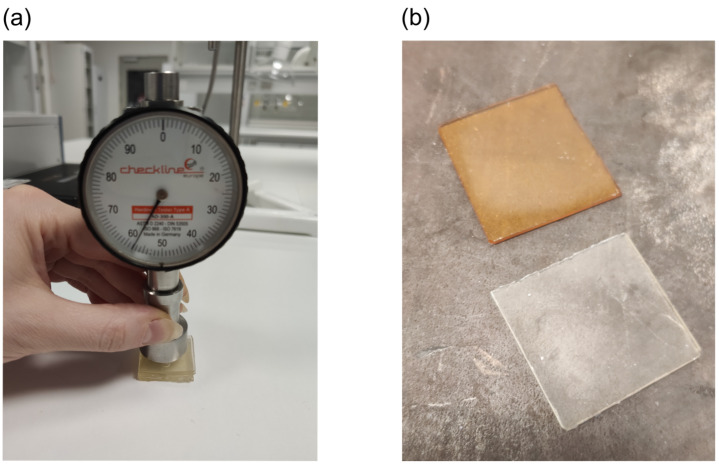
(**a**) Shore A measurement of cured, non-irradiated polymer material. (**b**) Polymer before (bottom) and after (top) post-manufacturing heating at 180 °C for 10 min.

The hardness of the polymer material was tested to evaluate if additional post-fabrication heating at a high temperature (180 °C) would be needed for full curing (monomer conversion) and stabilization of the remaining free radicals. The expectation was that the additional heating step would increase the stiffness of the polymer by increasing the crosslink density [49] and ultimately increase its hardness. As can be seen in Figure 6a, the material’s Shore A value was measured to be 57 ± 3, and after heating at 180 °C for 10 min, the value was 60 ± 6. No significant difference in hardness was observed. Moreover, an undesirable color formation was obtained after heating, which could worsen the optical properties of the material and hinder the leuco-dye reaction when irradiated. Hence, high-temperature heating after manufacturing was contraindicated. It is possible that yellowing of the sample was caused by the formation of benzil from benzoyl radicals, which were produced during the photopolymerization process of TPO [50].

#### 3.3.5. Rheology

**Figure 7:**
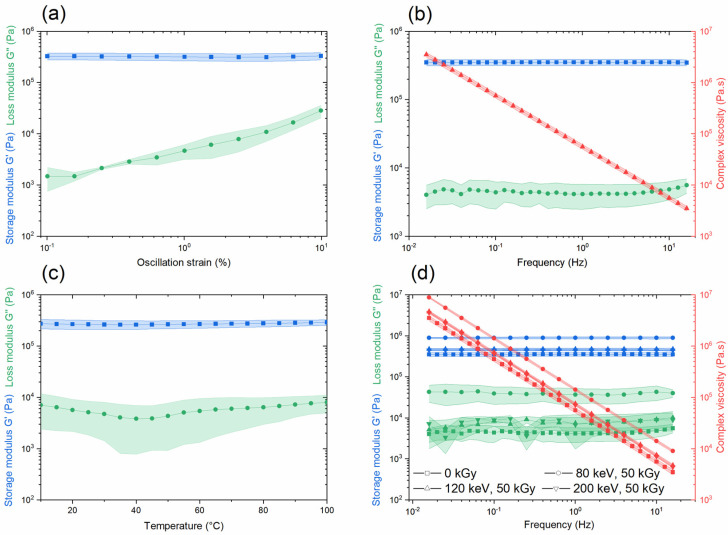
Rheology results: (**a**) amplitude sweep of non-irradiated polymer material with measurement performed in triplicate, (**b**) frequency sweep of non-irradiated polymer material with measurement performed in triplicate, (**c**) temperature sweep of non-irradiated polymer material with measurement performed in triplicate and (**d**) frequency sweep of irradiated polymer material with measurement performed in triplicate. Blue = storage modulus G′ (Pa); green = loss modulus G″ (Pa); red = complex viscosity (Pa·s). Error bar areas are filled under the curve.

In order to evaluate the structural and viscoelastic characteristics of the polymer material, amplitude and frequency sweeps were performed, as seen in Figure 7a,b, respectively. It can be noted that the storage modulus G′ was larger than the loss modulus G″ for all applied oscillation strains (Figure 7a), indicating that the elastic behavior of the material dominated over the viscous behavior. The permissible maximum strain frequency sweep measurement was chosen to be γ = 0.25%, and the value agreed with the estimation for cross-linked polymers of γ ≤ 1% [51,52]. Higher strains could cause an irreversible deformation, with a tendency toward viscous-like behavior. The variation in loss modulus values might be due to axial force changes or thickness variation of the sample introduced during the photocuring step. The differences in thickness could be a result of resin shrinkage during the photopolymerization reaction, which was caused by the reduction of distance between the monomers while shifting from longer double-bonds (van der Waals) to shorter covalent bonds [53].

Figure 7b shows the dependency of the storage and loss moduli on the frequency at a strain of 0.25%. Both G′ and G″ displayed almost parallel lines in the whole frequency range, showing the behavior of closely cross-linked polymers [51]. Moreover, G′ was high in relation to G″, and the loss tangent was $\tan\varphi =\frac{G^{”}}{G^{’}}\approx 0.01$. Even at lower frequencies, the values of G′ and G″ remained constant, showing a high structural strength (rigidity). From the polymer point of view, rigidity of the polymer matrix is required for efficient fluorescence emission of the immobilized dye [19,35].

The variations of the elastic and viscous moduli versus temperature for the non-irradiated polymer were also tested, and the curves are presented in Figure 7c. Here, G′ > G″ also remained valid over the whole temperature range. Above the glass transition temperature of the material, only a limited deformation of the polymer network is possible, depending on the length and density of the bonds. The material did not reach a liquid state even at high temperatures due to the strong chemical bonds that form the polymer network.

The frequency sweep of an irradiated polymer material was also tested, as presented in Figure 7d, for the samples irradiated with 50 kGy and electron beam energies of 80, 120 and 200 keV. Here as well, both the storage and loss moduli took the form of parallel lines in the chosen frequency range, with G′ always being much greater than G″. The irradiation process did not cause any significant structural changes to the material. A slight increase in G′ could be observed for the irradiated samples, which could be an indication of probable cross-linking reactions taking place at these very high doses [54] and a lack of chain scissions caused by the irradiation process [55,56]. All of this demonstrates the suitability of the chosen polymer matrix as a radiation dosimeter for low-energy electron beams.

#### 3.3.6. Water Affinity

**Figure 8:**
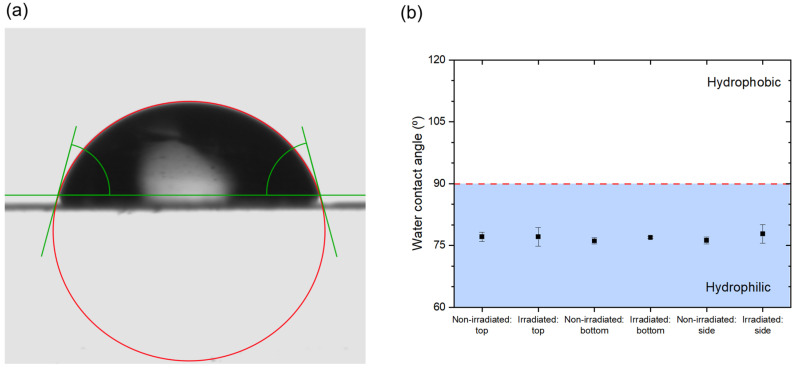
Water contact angle (WCA) measurements. (**a**) A single water drop placed on a polymer surface. The green horizontal line indicates a baseline, and the red circle is the circle of interest. The WCAs (left and right) are marked. (**b**) WCAs of non-irradiated and irradiated (200 keV, 30 kGy) polymer material (top, bottom and side). The red dashed line marks a 90° angle where the transition from hydrophilic to hydrophobic properties takes place [57].

Determination of the wettability of the polymer samples is extremely important for fluorescence microscopy measurements when using an immersion medium, because an inadequate choice of a surrounding medium might result in sample swelling or altering of its properties. As a result, additional uncertainties in fluorescence intensity measurements would be introduced. Additionally, when air is utilized as an imaging medium, the ability of the microscope objective to gather light and resolve fine details is limited by its numerical aperture, which in practice cannot be more than 1. However, by increasing the imaging medium’s refractive index to, for example, 1.33 for water, it is possible to obtain a higher numerical aperture, and ultimately, more details can be distinguished in a sample. Hence, we determined the water affinity of the polymer surfaces by measuring the water contact angle (WCA) as shown in Figure 8a. The WCA was measured for both the non-irradiated and irradiated (200 keV, 30 kGy) material, and the average values are presented in Figure 8b. It can be seen that none of the values exceeded 90°, indicating that the surfaces were hydrophilic [57] and had a high affinity with water. There was no significant difference between the top, bottom and side of the sample, indicating uniformity of the manufactured samples. Such a hydrophilic polymer network is similar to a hydrogel, which exhibits a swelling ability in water but will not dissolve when immersed in it [58]. Moreover, the affinity to water did not change after irradiation, which is sometimes introduced deliberately for other polymers by using electron beam irradiation [59]. Based on the obtained results, it is clear that the use of water immersion objectives should be avoided. It also suggests that the polymer material should be stored in a low-humidity environment.

### 3.4. Fluorescence Signal

#### 3.4.1. Fluorescence from the Dye Dissolved in a Liquid and Solid

**Figure 9:**
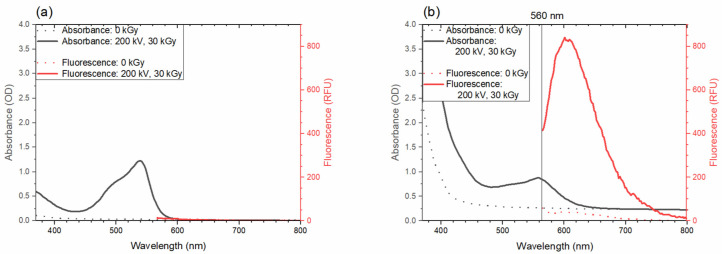
Absorbance and fluorescence spectra of the dye placed in: (**a**) a liquid solution of acetonitrile and (**b**) a polymer (solid) sample. Absorbance spectra are depicted as black solid and dashed lines for irradiated and non-irradiated samples, respectively. Fluorescence spectra are presented as red solid and dashed lines for irradiated and non-irradiated samples, respectively.

As a proof of concept, the absorbance and fluorescence spectra of the dye dissolved in a liquid and a solid sample were obtained, as can be seen in Figure 9a,b, respectively. The non-irradiated acetonitrile solution did not absorb or fluoresce, as expected, as is shown in the dashed black and red curves in Figure 9a. Then, once irradiated with a 200 keV e-beam to 30 kGy, an absorption peak with a maximum at around 555 nm was present, but there was no fluorescence emission (solid lines of the same figure). This observation agrees with the theory that triphenylmethane dyes need to be held in some fixed configuration when excited [35], and this was supported by the observation that after placing the dye in a stiff polymer matrix, a fluorescence signal with a peak at around 600 nm was detected, as presented in Figure 9b (solid red line). Additionally, a slight increase in the fluorescence signal for the non-irradiated sample was observed (dashed red line), which might have been due to the background absorbance of the sample at the excitation wavelength [60]. At the peak fluorescence value, the fluorescence intensity of the non-irradiated sample was 5% of the value of the sample irradiated with 30 kGy. This observation may have an influence on the dose-dependent fluorescence signal and the assessment of the dose, and it should be investigated in a separate study.

#### 3.4.2. Absorbance and Fluorescence Spectra from an 80 keV E-Beam-Irradiated Polymer

**Figure 10:**
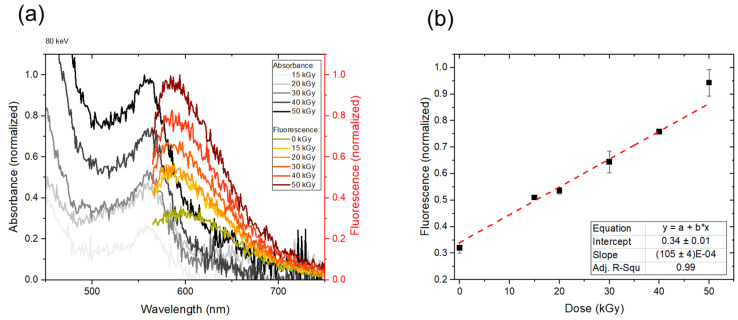
(**a**) Absorbance (grayscale) and fluorescence (red scale) spectra of polymer material irradiated with 80 keV electron beam of different doses. The spectra were normalized to the maximum values of the 50 kGy sample. (**b**) Peak fluorescence intensity versus dose for the polymer irradiated with 80 keV electron beam. The data points were fitted with a linear function of the parameters presented in the Table.

The excitation and emission spectra of the dosimeter material were measured for the samples (Type 3) irradiated with an 80 keV e-beam to doses of 15, 20, 30, 40 and 50 kGy, as shown in Figure 10a. The absorbance and fluorescence peak values increased with the absorbed dose. It should be noted that the penetration depth of the 80 keV e-beam is very small, of the order of 60 µm in water [2], and so the material needs to have good sensitivity. The noise present in the spectra may have its origin in a very high resolution (1 nm) set for the measured signal while keeping the excitation light bandwidth comparatively broad (5 nm). The background subtraction of the well plate and normalization process also introduced unwanted signal fluctuations. A possible way to reduce these would be to perform additional measurements, which could average out the noise.

The peak fluorescence intensity at 600 nm increased linearly with the dose as shown in Figure 10b, demonstrating the proof of concept of the dosimeter material. The trend is in agreement with the theory that at low concentrations of analyte, the emission intensity is proportional to its concentration [61]. However, a significant part of the fluorescence signal could also be observed for a non-irradiated material, introducing a non-zero background fluorescence signal. This would need to be subtracted from the fluorescence intensity measurements for correct dose estimation. However, this will cause the signal-to-noise ratio to decrease, and a part of the meaningful information might be lost. Another possibility, therefore, would be to include the background signal in the calibration of the material and thereby correct for the introduced offset. A following paper will describe the fluorescence imaging results, including the background information concerns.

## 4. Discussion

The development of a polymer material for its use as a radiofluorogenic dosimeter consisted of several interrelated steps, and only careful evaluation of each of them allowed for the choice of a final material with the necessary characteristics needed for fluorescence imaging using confocal laser scanning microscopy.

In terms of composition, the choice of a proper solvent for the radiation-sensitive dye was important, and acetonitrile showed the best performance. The dose-dependent fluorescence intensity is a function of the dye concentration, so any errors introduced already at this stage would propagate further and cause artifacts such as shadowing and saturation problems in fluorescence microscopy imaging.

During the selection of a host polymer, a compromise between rigidity and diffusion had to be met for an efficient reaction of the leuco-dye. Both acrylates and methacrylates were initially considered as candidates. However, the photopolymerization rates for methacrylates were much lower due to the stabilizing effect of the α-methyl group [62]. The functionality of a monomer was also a deciding factor, as higher functionalities led to higher reaction rates and faster vitrification. However, a higher cross-link density lowered the conversion, so more of the uncured monomer remained trapped between the cross-links [63]. Higher functionality of a monomer also means its viscosity is greater, which limits the easy handling of a solution. Therefore, lower functionalities were chosen, which due to slower vitrification enabled in-depth curing with less shrinkage and enhanced mobility of the system [64]. The length and nature of a spacer group were also considered, with a conclusion of a not-too-bulky and long spacer group between unsaturations. Poly(propylene glycol) diacrylate turned out to be an excellent candidate for the host polymer, but similar monomers could be also considered.

The photopolymerization process is responsible for the quality and uniformity of the produced samples. Hence, the lifetime of used silicone molds was limited to 2 months of use, corresponding to approximately 100 curing procedures. Even though the attempted use of 3D-printed molds was not successful here, they presented a great potential for a new method of sample production by using, for example, highly flexible 3D printing resins.

The polymer’s glass transition temperature, measured to be −44.45 °C, indicated that the material remained in a rubbery state at room temperature. Low T_g_ is also a sign of a higher cure extent [65]. Such an elastic material could be compared by its hardness of 57 ± 3 Shore A to a pencil eraser, indicating both toughness and pliability. The domination of elasticity was confirmed in the rheology measurements, where the elastic portion (G′) was around 100 times greater than the viscous one (G″) in the chosen frequency range, a feature similar to thermosets and closely cross-linked materials. Additionally, high thermal stability (up to 350 °C) was achieved in an inert atmosphere. This would be lowered once oxygen is introduced into the system. It is a great advantage that none of these features were influenced by the absorbed dose, meaning that the radiation-induced defects were not the most significant obstacles.

For fluorescence imaging, the assessment of the refractive index and the water affinity is crucial. Thus, a refractive index matching medium cannot be water due to the hydrophilic properties of the polymer, and it should be chosen so that *n* = 1.47 ± 0.01. The refractive index was measured at 1300 nm, and it might be somewhat greater at the excitation wavelength of 555 nm due to chromatic dispersion [48].

All of the above had an impact on the observed fluorescence signal; a molecule non-fluorescent in liquid became fluorescent once embedded in a rigid polymer matrix. The solid state polymer restricted the motion of the benzene groups of the leuco-dye. Irradiation with the low-energy electron beam at high doses caused the fluorescence signal to increase linearly with the dose, as expected, for increasing concentration of excited fluorophores. The background signal will have to be taken into account during fluorescence imaging, which can be solved by either its subtraction or inclusion in calibration. The first one may limit the dynamic range and lower the signal-to-noise ratio, whereas the second might complicate the readout procedure.

There exists a demand for the development of new radiofluorogenic dosimeters, especially in the medical dosimetry field [66,67,68,69,70]. Typically, radiofluorogenic gels are investigated, which show a linear increase in fluorescence intensity with the dose (Gy) but often lack system stability due to diffusion of the radiation products, oxygen interference and dose rate dependency. Our polymer material, even though intended to work with much higher doses, brings advantages in terms of mechanical and thermal stability, as well as the possibility of 3D signal detection when imaged using confocal laser scanning microscopy. The limitations of our study, such as low measurement reproducibility and a limited number of tested compositions and characterization techniques, should be accounted for in future studies of the radiofluorogenic polymer dosimeter.

## 5. Conclusions

The present study characterized a novel polymer material as a dosimeter candidate for high-dose, low-energy electron beam irradiations widely used for product surface sterilizations. The current lack of a dosimetry system being able to resolve the information about the surface dose might be overcome with the proposed solid state radiofluorogenic polymer material, which responds linearly to the absorbed dose and shows properties not harmed by it. This material is the first of its kind to deliver information about the absorbed dose distribution in 3D, which is to be measured using confocal microscopy.

The properties of the proposed polymer material were analyzed in order to establish the required characteristics for a reliable solid state dosimeter. Acetonitrile showed the best performance in dissolving the leuco-dye. Improved rigidity was achieved by using poly(propylene) glycol diacrylate as a host polymer. The thermal decomposition of the polymer was measured by TGA to occur at 350 °C and was not influenced by the irradiation process. The glass transition temperature determined from DSC measurement was −44.45 ± 1.35 °C with no changes after irradiation. A 57 ± 3 Shore A hardness was determined, and high-temperature heating was not needed for improved conversion. The refractive index of the material, evaluated using OCT, was equal to *n* = 1.47 ± 0.01. A closely cross-linked polymer, with a dominating storage modulus (G′ > G′′) in the analyzed frequency and temperature range, was found. A water contact angle of less than 90° was measured, indicating a hydrophilic material. The fluorescence intensity of the dye, which was not seen for the liquid system, increased linearly with the absorbed dose, although it exhibited a non-zero background signal. To conclude, the polymer demonstrated promising characteristics for use as a dosimeter in low-energy electron beams. The readout of the fluorescence signals will be further investigated in a following paper.

## Figures and Tables

**Table 1:** Specification of the irradiation process parameters.

Nominal Irradiation Dose (kGy)	Voltage (kV)		
	80	120	200
	Beam Current (mA)		
10	1.746	-	-
15	2.619	-	-
20	3.492	-	-
30	5.238	3.065	4.639
40	6.894	-	-
50	8.730	5.108	7.733

## Data Availability

The data presented in this study are available on request from the corresponding author.
